# Treatment with the Bacterial Toxin CNF1 Selectively Rescues Cognitive and Brain Mitochondrial Deficits in a Female Mouse Model of Rett Syndrome Carrying a MeCP2-Null Mutation

**DOI:** 10.3390/ijms22136739

**Published:** 2021-06-23

**Authors:** Chiara Urbinati, Livia Cosentino, Elena Angela Pia Germinario, Daniela Valenti, Daniele Vigli, Laura Ricceri, Giovanni Laviola, Carla Fiorentini, Rosa Anna Vacca, Alessia Fabbri, Bianca De Filippis

**Affiliations:** 1 Center for Behavioral Sciences and Mental Health, Istituto Superiore di Sanità, 00161 Rome, Italy; chiara.urbinati@iss.it (C.U.); livia.cosentino@iss.it (L.C.); viglidaniele@gmail.com (D.V.); laura.ricceri@iss.it (L.R.); giovanni.laviola@iss.it (G.L.); 2 Department of Cardiovascular, Endocrine-Metabolic Diseases and Aging, Istituto Superiore di Sanità, 00161 Rome, Italy; elena.germinario@iss.it (E.A.P.G.); alessia.fabbri@iss.it (A.F.); 3 Bioenergetics and Molecular Biotechnologies, Institute of Biomembranes, National Council of Research, 70126 Bari, Italy; d.valenti@ibbe.cnr.it (D.V.); r.vacca@ibiom.cnr.it (R.A.V.); 4 Association for Research on Integrative Oncology Therapies (ARTOI), 00165 Rome, Italy; carla.fiorentini@artoi.it

**Keywords:** Rett syndrome, mouse models, cognition, energy metabolism, mTOR, mitochondria, Rho GTPases, behaviour

## Abstract

Rett syndrome (RTT) is a rare neurological disorder caused by mutations in the X-linked *MECP2* gene and a major cause of intellectual disability in females. No cure exists for RTT. We previously reported that the behavioural phenotype and brain mitochondria dysfunction are widely rescued by a single intracerebroventricular injection of the bacterial toxin CNF1 in a RTT mouse model carrying a truncating mutation of the *MeCP2* gene (MeCP2-308 mice). Given the heterogeneity of *MECP2* mutations in RTT patients, we tested the CNF1 therapeutic efficacy in a mouse model carrying a null mutation (MeCP2-Bird mice). CNF1 selectively rescued cognitive defects, without improving other RTT-related behavioural alterations, and restored brain mitochondrial respiratory chain complex activity in MeCP2-Bird mice. To shed light on the molecular mechanisms underlying the differential CNF1 effects on the behavioural phenotype, we compared treatment effects on relevant signalling cascades in the brain of the two RTT models. CNF1 provided a significant boost of the mTOR activation in MeCP2-308 hippocampus, which was not observed in the MeCP2-Bird model, possibly explaining the differential effects of CNF1. These results demonstrate that CNF1 efficacy depends on the mutation beared by MeCP2-mutated mice, stressing the need of testing potential therapeutic approaches across RTT models.

## 1. Introduction

Rett syndrome (RTT; OMIM #312750) is an early-onset neurologic disorder that manifests in a wide range of symptom severities [1]. RTT is classified as a rare X-linked dominant disorder affecting mostly females (1 in 10,000 female births), since males are more severely affected, and rarely survive infancy [2]. In its classical form, RTT patients show an apparently normal psychomotor development until 6–18 months of age, when a characteristic neurological regression occurs, with loss of acquired motor, communicative, and cognitive skills, and development of autistic-like features [1]. In addition to the extremely impairing neurobehavioural issues, multisystem comorbidities occur during patients’ lifespans, that can last up to 50–70 years [1].

Most classic RTT cases are caused by *de novo* mutations in the X-linked gene *methyl-CpG-binding protein 2* (*MECP2*) [1]. Available data point to a crucial role of MECP2 in fundamental neurodevelopmental processes such as activity-dependent regulation of neuronal morphogenesis and plasticity. Defective mitochondrial energy production machinery and the resulting increased levels of free radicals are also emerging as relevant factors in the pathogenesis of RTT [3,4]. However, the molecular mechanisms leading from *MECP2* gene mutations to RTT symptomatology and progression have not been completely clarified. No effective therapy is currently available [5].

The discovery of the monogenic origin of RTT has led to the generation of different lines of transgenic mouse models in an attempt to cover the spectrum of *MECP2* mutations observed in RTT patients [6]. In fact, affected females show a wide variety of phenotypic alterations, with mutation status being a strong predictor of symptoms severity [6]. For instance, milder phenotypes have been associated with C-terminal deletions, a type of mutation which accounts for about 10% of RTT cases, whereas early truncating mutations are responsible for the most severe symptomatology [7]. These RTT mouse models, that closely mimic the clinical features of the human disorder, including the mitochondrial impairments described in patients [4,8], have proved fundamental to identify compounds with therapeutic potential for RTT [7]. Several laboratories have in fact demonstrated the reversibility of the RTT symptomatology through genetic and pharmacological approaches (reviewed in [9]).

In this context, we have reported that a single intracerebroventricular (icv) injection of a bacterial toxin, named cytotoxic necrotising factor 1 (CNF1), persistently rescues cognitive and motor impairments, and synaptic plasticity deficits in a RTT mouse model carrying a *MeCP2* late truncating mutation that reproduces the condition of milder RTT cases (MeCP2-308 mice) [10]. CNF1 treatment also restores the alterations in the activity of the mitochondrial respiratory chain (MRC) complexes and of ATP synthase, the molecular machinery responsible for the majority of cell energy production [11,12].

CNF1 is a bacterial protein from *Escherichia coli* that can permanently activate Rho GTPases, a family of molecules critically involved in the control of neuronal plasticity and cognition [13]. CNF1 brain inoculation in fact induces a rearrangement of cerebral actin cytoskeleton, enhances neurotransmission and synaptic plasticity and improves learning and memory performance in mice [14,15]. The great potential of such a therapeutic strategy is further highlighted by studies demonstrating that CNF1 administration also rescues the neurobehavioural abnormalities in a mouse model of Alzheimer’s disease, another disorder characterised by cognitive dysfunction [16]. Moreover, although no systematic studies aimed at understanding the time progression of CNF1 effects have been so far carried out, available data suggest that brain architecture and functionality in mice may be persistently modulated by a single icv administration of CNF1 [17]. No chronic dosing of CNF1, which might be expected to have side effects, may thus be required. These results clearly indicate that modulation of brain Rho GTPases by a single administration of CNF1 may constitute an innovative therapeutic approach for RTT. The pivotal role of Rho GTPases in the rescue of RTT symptoms we observed in CNF1-treated mice was in fact unequivocally confirmed by the total absence of effects on mice receiving, as a control treatment, the mutant protein CNF1 C866S (mCNF1), devoid of Rho enzymatic activity [10].

Given the heterogeneity of *MECP2* mutations in RTT patients, to test the robustness and generalisation of CNF1 therapeutic efficacy, the present study extended the investigation to a second RTT mouse model, characterised by a more severe phenotype, carrying a *MeCP2* null mutation modelling the large deletions found in 10% of RTT patients (MeCP2-Bird mice) [18]. MeCP2-mutated heterozygous female (Het) mice were used in all the experiments since they recapitulate the genetic and hormonal condition which more closely resembles that of RTT patients [19]. To verify whether the CNF1 treatment was as efficacious as in the MeCP2-308 mouse model in contrasting the RTT-like phenotype, a battery of behavioural tests was carried out in mice that received a single icv injection of CNF1 or mCNF1, as a control (Supplementary Figure S1). Treatment effects on MRC complex activities were also explored in MeCP2-Bird mouse brain, to verify whether the CNF1 was able to rescue these alterations as in the MeCP2-308 model [11,12]. CNF1 treatment effects on signalling cascades known to be altered in RTT brain were also compared in the two RTT models. A focus was made on the mammalian target of rapamycin (mTOR) pathway, a central regulator of a diverse array of cellular processes, including cell growth, proliferation, autophagy, translation, and actin polymerisation [20]. Increasing evidence in fact suggests that mTOR contributes to RTT pathogenesis, and its modulation may represent a promising therapeutic strategy against RTT [21,22,23].

## 2. Results

### 2.1. CNF1 Treatment Improves the Fear Memory Deficits in MeCP2-Bird Mice

**Figure 1:**
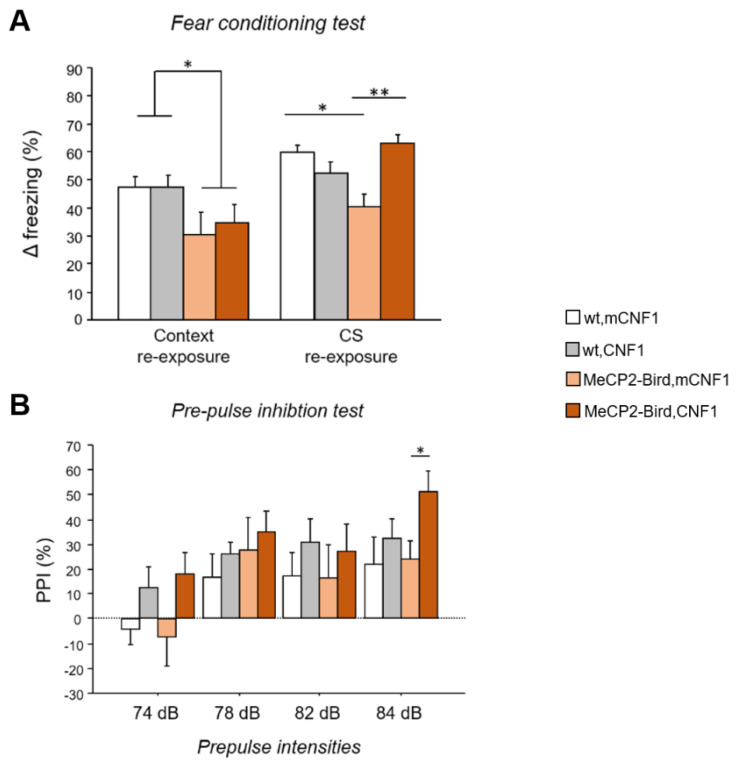
CNF1 treatment improves MeCP2-Bird cued fear memory deficits and increases pre-pulse inhibition in MeCP2-Bird mice. (**A**) Contextual and cued fear memory were evaluated by testing experimental mice in a fear conditioning task. The Δ freezing was calculated as an index of fear memory and obtained by subtracting the percent time spent freezing during baseline from percent time spent freezing during either context or conditioned acoustic stimulus (CS) re-exposure. Symptomatic MeCP2-Bird female mice treated with an inactive form of the bacterial toxin (mCNF1) showed impaired memory for the aversive context and the CS, as demonstrated by lower Δ freezing showed during context and CS re-exposure, compared to wild type controls (wt, mCNF1). Treatment with CNF1 restored wt-like levels of Δ freezing to the CS, without affecting memory for the aversive context. (**B**) Experimental animals’ sensorimotor gating abilities were evaluated exposing mice to the pre-pulse inhibition (PPI) paradigm, consisting in reduction in the startle response to an acoustic pulse that occurs when presented with a pre-pulse stimulus. MeCP2-Bird mice did not display PPI deficits. Treatment with CNF1 tended to increase the PPI reflex in both wt and MeCP2-Bird mice, reaching a significant effect only in MeCP2-Bird mice at a pre-pulse intensity of 84 dB. The percentage of PPI was calculated, for each pre-pulse intensity, as follows: 100-[(mean startle amplitude for pre-pulse + pulse trials / mean startle amplitude for pulse alone trials) × 100]. Mice for each condition were as follows: wt, mCNF1: 7; wt, CNF1: 7; MeCP2-Bird, mCNF1: 6; MeCP2-Bird, CNF1: 6. Data are mean ± SEM. Statistical significance was assessed using three-way mixed ANOVA and Tukey’s *post hoc* tests. *: *p* < 0.05; **: *p* < 0.01.

To verify whether the CNF1 treatment can rescue RTT cognitive deficits in MeCP2-Bird female mice, a fear conditioning (FC) protocol was applied [10]. In the contextual re-exposure phase, MeCP2-Bird mice showed lower levels of freezing compared to wild type (wt) controls (genotype: F_(1,22)_ = 6.606, *p* = 0.018; Figure 1A), suggesting the presence of defective fear memory. Treatment with CNF1 did not rescue the phenotype of RTT mice during aversive context re-exposure. When re-exposed to the conditioned acoustic stimulus (CS), MeCP2-Bird mice still showed lower levels of freezing compared to wt controls (*p* < 0.05 after *post hoc* comparison on the genotype ×treatment interaction: F_(1,21)_ = 16.716; *p* = 0.005; Figure 1A). Interestingly, the CNF1 treatment increased the freezing response of MeCP2-Bird mice selectively in the cued fear memory test (*p* < 0.01 after *post hoc* comparison on the genotype × treatment interaction, Figure 1A), thus restoring wt-like levels.

Importantly, no significant differences were found among genotypes in the percent time spent freezing at BL (genotype: F_(1,21)_ = 0.086; *p* = 0.772, data not shown), supporting the interpretation that cognitive, rather than motor impairments, are responsible for the aberrant behaviour displayed by MeCP2-Bird mice during the conditioning test, and for CNF1 treatment effects.

### 2.2. CNF1 Treatment Increases Pre-Pulse Inhibition in MeCP2-Bird Mice

To evaluate the sensorimotor gating of experimental mice, which is used as a measure of cognitive function in neuropsychiatric disorders [24], the pre-pulse inhibition (PPI) test was carried out. MeCP2-Bird mice did not display sensorimotor gating deficits, as demonstrated by the absence of significant differences from wt controls in the %PPI at different pre-pulse intensities (74, 78, 82, 84 dB; Figure 1B). Treatment with CNF1 displayed a tendency towards increasing %PPI in both wt and MeCP2-Bird mice (treatment: F_(1,22)_ = 3.576, *p* = 0.072). Interestingly, the treatment provided a significant improvement of MeCP2-Bird mice performance at 84 dB pre-pulse intensity (*p* < 0.05 after *post hoc* comparison on pre-pulse intensities × genotype × treatment interaction: F_(3,66)_ = 0.665, *p* = 0.576; Figure 1B). The analysis of acoustic startle response (ASR) revealed a reduction in the ASR of MeCP2-Bird mice in comparison to wt controls (genotype: F_(1,22)_ = 10.512; *p* = 0.004) and CNF1 did not affect this parameter (Supplementary Figure S2).

### 2.3. CNF1 Treatment Does Not Rescue the Motor Deficits and the General Health Status of MeCP2-Bird Mice

**Figure 2:**
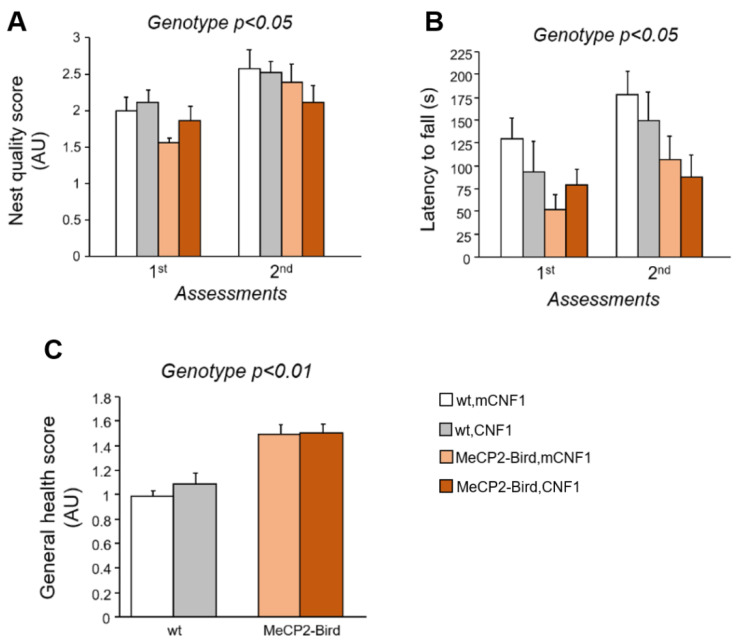
Treatment with CNF1 does not rescue the motor deficits and the impaired general health status of symptomatic MeCP2-Bird female mice. (**A**) Coordinated and purposeful forepaw use was evaluated twice throughout the experimental schedule by the means of the nest building task. Nest quality was assessed by a trained observer blind to mouse genotype and treatment and measured in average units (AU). Symptomatic MeCP2-Bird female mice treated with an inactive form of the bacterial toxin (mCNF1) showed impaired nest building capacity relative to wild type (wt) controls, as demonstrated by the lower nest quality score. CNF1 treatment did not affect their performance. (**B**) Motor learning and coordination was evaluated through the accelerating rotarod task, that was repeated two times during the experiment. Symptomatic MeCP2-Bird females showed reduced motor coordination abilities, as represented by the lower latencies displayed to fall from the rotating rod. The graph represents the average latency out of three consecutive trials/assessment. (**C**) The general health status was evaluated throughout the experimental schedule by a trained observer blind to animals’ genotype and treatment. The average score of the five assessments is measured in AU and represented in the figure. Symptomatic MeCP2-Bird mice displayed impaired general health conditions compared to wt controls. Treatment with CNF1 was not able to rescue any of these behavioural impairments. Mice for each condition were as follows: wt, mCNF1: 7; wt, CNF1: 7; MeCP2-Bird, mCNF1: 5–6; MeCP2-Bird, CNF1: 6. Data are mean ± SEM. Statistical significance was assessed using three-way mixed ANOVA.

To evaluate the efficacy of CNF1 treatment on the coordinated and goal-directed use of forelimbs, that is severely impaired in RTT patients and mouse models [25], the nest building abilities of MeCP2-Bird mice were evaluated on the 6th and 22nd days of the experimental schedule (Supplementary Figure S1). All the experimental mice improved their ability to manipulate the nest material along the two assessments (day: F_(1,22)_ = 16.780; *p* = 0.005). However, nest building capacity was impaired in MeCP2-Bird mice compared to wt controls (genotype: F_(1,22)_ = 4.505, *p* = 0.045; Figure 2A), thus substantiating the presence of altered forepaws coordination in this RTT model. CNF1 treatment did not ameliorate the quality of the nests built by RTT mice in either assessment.

Treatment effects on motor coordination and learning abilities were further evaluated by the means of the accelerating rotarod test. Predictably, MeCP2-Bird mice showed a reduced latency to fall from the rod with respect to wt controls (genotype: F_(1,21)_ = 5.299; *p* = 0.032, Figure 2B). CNF1 treatment did not rescue the impaired motor coordination skills of MeCP2-Bird mice.

The evaluation of experimental animals’ general health (GH) status was performed five times throughout the experimental schedule. The analysis of the GH score confirmed that MeCP2-Bird mice displayed worse health conditions compared to wt controls throughout the experiment (genotype: F_(1,22)_ = 10.943; *p* = 0.003; Figure 2C). No effects of the treatment with CNF1 were found. Figure 2C represents the average of the five assessments. Single day scores are reported in Supplementary Figure S3.

### 2.4. CNF1 Treatment Rescues the Defective Activity of Mitochondrial Respiratory Chain Complexes I, II and V in MeCP2-Bird Mice

**Figure 3:**
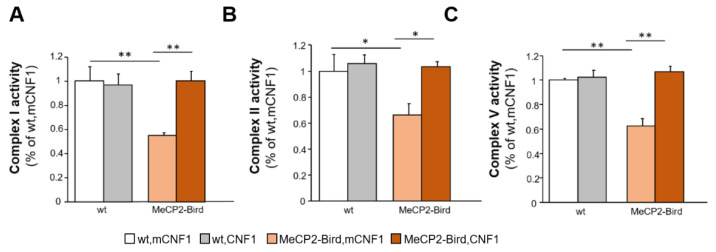
Treatment with CNF1 normalises the activity of mitochondrial respiratory chain complexes in symptomatic MeCP2-Bird mouse cortices. The activity of the mitochondrial respiratory chain (MRC) complexes I (**A**), II (**B**) and V (**C**) were measured spectrophotometrically in mitochondrial membrane-enriched fractions of experimental animals’ brain cortices. Symptomatic MeCP2-Bird female mice treated with an inactive form of the bacterial toxin (mCNF1) displayed reduced MRC complexes activity relative to wild type controls (wt, mCNF1). CNF1 restored wt-like levels of activity in treated MeCP2-Bird mice. Complex activities are expressed as percentage of the activity measured in wt, mCNF1 (set equal to one). Mice for each condition were as follows: wt, mCNF1: 3; wt, CNF1: 4; MeCP2-Bird, mCNF1: 4; MeCP2-Bird, CNF1: 4. Data are mean ± SEM obtained from three independent experiments. Statistical significance was assessed using two-way ANOVA and Tukey’s *post hoc* tests. *: *p* < 0.05; **: *p* < 0.01.

We previously demonstrated that CNF1 treatment rescues the defective mitochondrial functionality in MeCP2-308 mouse brains [11]. Based on this, we hereby evaluated whether CNF1 might prove beneficial in rescuing the mitochondrial deficits in MeCP2-Bird mouse cortices. We focused on the activation of the MRC complexes I, II and V since they were found impaired in MeCP2-Bird mouse brain [26]. We confirmed that complexes I (*p* < 0.01 after *post hoc* comparison on genotype × treatment interaction: F_(1,11)_ = 9.720, *p* = 0.009; Figure 3A), II (*p* < 0.05 after *post hoc* comparison on genotype × treatment interaction F_(1,11)_ = 3.206, *p* = 0.101; Figure 3B), and V (*p* < 0.01 after *post hoc* comparison on genotype × treatment interaction: F_(1,11)_ = 16.328; *p* = 0.002, Figure 3C) display a significantly reduced activity in MeCP2-Bird brain mitochondria. In line with the beneficial effects exerted in MeCP2-308 mice [11], CNF1 treatment increased the activity of all defective MRC complexes in MeCP2-Bird mice (complex I: *p* < 0.01; Figure 3A; complex II: *p* < 0.05; Figure 3B; complex V: *p* < 0.01; Figure 3C), thus restoring wt-like activity values.

### 2.5. CNF1 Treatment Provides a Significant Boost of mTOR Activation in MeCP2-308 Mouse Hippocampus, Which Is Not Recapitulated in the Bird Model

To shed light on the molecular mechanisms underlying the partial recovery observed in MeCP2-Bird mice and the differential effects provided by CNF1 in the two RTT models, we analysed the activation status of mTOR based on previous studies demonstrating that mTOR signalling pathway is crucially involved in RTT pathophysiology [21,22,23].

**Figure 4:**
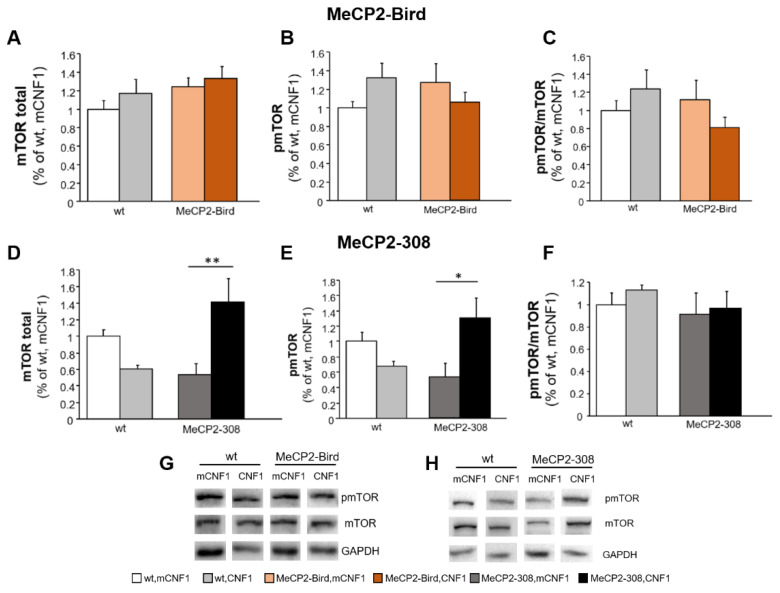
CNF1 treatment activates mTOR in the hippocampus of MeCP2-308 mice, but not in the MeCP2-Bird model. Total and phosphorylated (active) mTOR levels were evaluated by the means of Western blot analyses in hippocampi dissected from the brains of MeCP2-Bird, MeCP2-308 mice, and wild type (wt) littermates, treated with the recombinant (inactive, mCNF1) or active form of the bacterial toxin CNF1. (**A**–**C**) mTOR total or phosphorylated (pmTOR) levels and pmTOR/mTOR ratio did not significantly differ among groups in the MeCP2-Bird cohort. (**D**–**F**) CNF1 significantly increased both total and pmTOR levels in MeCP2-308 mice hippocampi but not the pmTOR/mTOR ratio**.** mTOR and pmTOR levels are normalised to total glyceraldehyde-3-phosphate dehydrogenase (GAPDH) contents and expressed as a proportion of those of wt, mCNF1 mice. (**G**,**H**) Immunoblots are examples from one animal of each experimental group. Mice for each condition were as follows: wt, mCNF1: 4 and 12; wt, CNF1: 4 and 12; MeCP2-Bird, mCNF1: 12; MeCP2-Bird, CNF1: 12; MeCP2-308, mCNF1: 4; MeCP2-308, CNF1: 4. Data are mean ± SEM. Statistical significance was assessed using two-way ANOVA and Tukey’s *post hoc* tests. *: *p* < 0.05; **: *p* < 0.01.

The total expression levels of mTOR did not significantly differ from wt controls in either MeCP2-Bird or MeCP2-308 mice (Figure 4A,D). Interestingly, CNF1 significantly increased mTOR levels in MeCP2-308 mice (*p* < 0.01 after *post hoc* comparison on genotype × treatment interaction: F_(1,12)_ = 15.892, *p* = 0.002; Figure 4D). A similar profile was observed for the levels of phospho-mTOR at Ser 2448 (pmTOR), which provide an index of mTOR activation. Indeed, no differences were found among groups in the MeCP2-Bird model (Figure 4B). Conversely, in the MeCP2-308 cohort, treatment with CNF1 significantly increased pmTOR levels compared to mutant mice treated with vehicle (*p* < 0.05 after *post hoc* comparison on genotype × treatment interaction: F_(1,12)_ = 10.894, *p* = 0.006; Figure 4E). Statistical analyses of pmTOR/mTOR ratio revealed no significant genotype or treatment effects in either of the two strains (Figure 4C,F).

### 2.6. CNF1 Treatment Does Not Affect Expression Levels of Mitochondrial and Synaptic Markers in RTT Mouse Brain

We also investigated CNF1 effects on mitochondrial and synaptic markers in the hippocampus of the two strains, since available literature suggests they represent the most affected domains by this treatment strategy [27,28].

Western blot analyses revealed that the expression levels of Tom20, a central component of the translocase of the outer membrane (Tom) of the mitochondria and the main entry gate for nuclear-encoded proteins [29], was significantly increased in mCNF1-treated MeCP2-Bird mice compared to wt controls (*p* < 0.05 after *post hoc* comparison on genotype × treatment interaction: F_(1,42)_ = 3.154, *p* = 0.083; Figure S4A). CNF1 did not significantly modulate Tom20 levels in MeCP2-Bird brains (Figure S4A). Conversely, no significant differences were found in Tom20 levels between MeCP2-308 and wt control mice (Figure S4C). Interestingly, treatment with CNF1 had the effect of reducing Tom20 expression (treatment: F_(1,30)_ = 4.401, *p* = 0.044; Figure S4C) only in MeCP2-308 mice.

We also investigated the levels of the dendritic spine protein spinophilin, which is particularly abundant in dendritic spines where it is involved in the regulation of spine density and functioning [30]. Both strains showed significantly higher levels of spinophilin in the hippocampus compared to wt (MeCP2-Bird—*p* < 0.05 after *post hoc* comparison on the genotype × treatment interaction: F_(1,18)_ = 12.236, *p* = 0.003; MeCP2-308—genotype: F_(1,24)_ = 5.313, *p* = 0.030; Figure S5A,B). Treatment with CNF1 did not normalise this phenotype in either RTT model (Figure S5A,B).

## 3. Discussion

The present study provides evidence that the extent of the effects of the bacterial protein CNF1 in contrasting RTT-like behavioural alterations depends on the mutation beared by the MeCP2 mouse line. Treatment with CNF1 in fact selectively improved the cognitive performance in mice carrying a MeCP2-null mutation, without ameliorating the general health status and the motor function, as the same treatment did in the MeCP2-308 mice carrying a late truncating mutation [10,11]. Interestingly, the cognitive beneficial effects observed in MeCP2-Bird mice were accompanied by a rescue of the defective activity of mitochondrial complexes at brain level, paralleling the effects previously observed in the MeCP2-308 model [11,12]. However, in the brain of MeCP2-Bird mice, the CNF1 treatment failed to provide the same boost to mTOR pathway activation outlined in the MeCP2-308 strain, which possibly accounts for the lack of widespread efficacy in this RTT mouse model.

From a translational point of view, the findings of the present study support a precision medicine approach because of the differential effects provided by CNF1 in the two RTT models. These results in fact suggest that the CNF1 treatment efficacy may be maximal in patients bearing late truncating mutations.

In terms of preclinical RTT studies, the present findings strongly stress the need to test the robustness of potential therapeutic approaches for RTT across different MeCP2-mutated models. Indeed, although a high number of mouse models are currently available which do constitute a precious tool for the RTT research field, systematic comparisons among models carrying different *MeCP2* mutations (including different genetic backgrounds) are still missing and very few compounds have been tested in more than one model so far [31,32,33]. A better understanding of the molecular mechanisms underlying the genotype–phenotype correlations in models carrying *MeCP2* mutations would certainly provide relevant information concerning the potential generalisation of treatments or the need to develop personalised approaches.

The search for CNF1-induced molecular effects that could explain a different treatment efficacy in the two models allowed us to determine that the activation of the mTOR signalling pathway was increased by the CNF1 treatment in the MeCP2-308 mouse brain only. Although further studies are needed to verify whether this specific effect actually accounts for the wider beneficial effects we observed in the MeCP2-308 mouse model, several lines of evidence support this possibility. In fact, the reduced activation of the mTOR signalling pathway was confirmed in MeCP2-mutated human neurons [21,34] and the activation of mTOR was found to improve abnormal phenotypes in MeCP2 mutant mice, RTT patient-derived neurons, and human embryonic stem cell-derived neurons with MECP2 mutation [34,35,36]. Furthermore, treatments exerting opposite effects on mTOR activity were found to either ameliorate or induce RTT-like neuronal phenotypes [37]. Taken together, this evidence supports a critical involvement of mTOR dysregulation in RTT pathogenesis and underlines its potential relevance as a therapeutic target for RTT.

Although currently we do not have specific explanations regarding the mechanisms through which CNF1 exerts different effects on the mTOR signalling pathway in the two RTT models, we can speculate that in the MeCP2-308 model, the truncated form of MeCP2 may still retain partial functionality, that might be fundamental for the CNF1 effects on mTOR to occur. The posttranslational modification status of the truncated MeCP2 protein may for instance still be modified by the CNF1 treatment, possibly leading to an improvement in some MeCP2 functions [38]. Given that MeCP2 was found to regulate mTOR activity through miR-199a processing facilitation, it is, for instance, conceivable that CNF1 may restore the capability of the truncated MeCP2 to associate with the miRNA Microprocessor Drosha Complex [37]. On the other hand, the CNF1 recovering effect on mitochondrial function without mTOR activation also suggests that CNF1-induced rescue of MRC deficient activities in RTT mouse brain may involve other mechanisms. Further studies are certainly needed to dissect the mechanisms involved in CNF1 therapeutic effects.

Interestingly, we found that the CNF1 improved, in MeCP2-Bird Het mice, the performance in the fear conditioning test and, to a certain degree, also in the PPI, suggesting a selective treatment effect on cognitive dysfunction in this RTT mouse model. Importantly, this was accompanied by restoration of mitochondrial complex activities, suggesting that the rescue of the brain energy reservoir may be fundamental to improve cognitive function in RTT. Indeed, maintaining a steady balance between nutrient supply and energy demand is an essential mechanism for preserving higher brain functions under different conditions [39,40,41].

It is, however, worth noting that in the present study we did not recapitulate the enhanced PPI previously reported in symptomatic MeCP2-null heterozygous female mice [42]. One possible explanation for the discrepancy between present results and those from Samaco and colleagues is related to the different age of the animals. In fact, according to Samaco, PPI alterations are not yet evident at 20 weeks of age and would appear at later stages of the disease (52 weeks of age). We cannot thus exclude that the younger age of our mice (<36 weeks) may have contributed to determine the lack of PPI defects in MeCP2-Bird mice. The genetic background of mice may also account for the different results. Samaco et al. in fact tested two mouse models carrying the MeCP2 null mutation on mixed genetic backgrounds (FVB/*n* × 129S6/SvEv and 129S6/SvEv × C57BL/6), while our animals are on a C57BL/6Jbackground.

Contrary to our expectations, CNF1 treatment-induced improvements in mitochondrial complex activities were not accompanied by the rescue of health conditions and motor dysfunction in MeCP2-Bird mice. A similar effect was previously observed in MeCP2-308 mice receiving a 10-day-long treatment with metformin, the first-line therapy for type 2 diabetes [41]. Taken together, these data, whereas not challenging the pathogenic role of brain mitochondrial dysfunction for RTT, suggest that selectively targeting these alterations may not be the most promising therapeutic strategy for this disorder at an advanced stage of the disease, when alterations deriving from an aberrant energy metabolism during neurodevelopment, may not be reversible any longer. Several studies have in fact demonstrated that the appearance of mitochondrial dysfunction in RTT mouse models [3], which can be expected to impact neurogenesis and neural circuits rearrangement [43,44], precedes the establishment of overt behavioural symptoms. Better outcomes may thus be achieved, for RTT pathophysiology, with earlier administrations of molecules targeting brain mitochondrial dysfunction [45], starting before symptoms onset.

The present results must be interpreted in light of some limitations. First of all, MeCP2-Bird mice of 5–10 months were enrolled in the study. This age range, although variable, was chosen since MeCP2-Bird heterozygous female mice are expected to start showing peculiar symptomatology after three months of age and live over 12 months [6]. In fact, although we cannot completely exclude that the age range could have influenced the behavioural data, RTT-like behavioural alterations were present in all the paradigms that were applied, with the exception of the PPI.

Another potential limitation concerns the lack of modification in the pmTOR/mTOR ratio in the brain of MeCP2-308 mice. We do in fact report that a general increase of both the molecule and of its active/phosphorylated form occurs in the brain of MeCP2-308 mice treated with CNF1, that is not present in the Bird model. Although we believe it is reasonable to consider the increased abundance of phosphorylated mTOR as a reliable indicator of its activation state, since this is accompanied by a similar increase of its total levels, further studies aimed at testing CNF1 effects on its downstream targets are needed to verify this hypothesis.

## 4. Materials and Methods

### 4.1. Subjects

The experimental subjects were heterozygous (Het) female mice and wild type (wt) littermates from two strains: the MeCP2-Bird model [B6.129P2(C)-Mecp2 tm1.1Bird/J, stock number: 003890] [18] and the MeCP2-308 model [B6.129S-MeCP2tm1Heto/J, stock number: 005439] [46]. Both strains were purchased from the Jackson Laboratories (Bar Harbor, ME, USA) and backcrossed to C57BL/6J mice for at least 12 generations in our animal facility.

Experimental animals for each RTT model were obtained by mating Het with wt males. Offspring were weaned at postnatal day 25 and housed according to sex in groups of 2–3 in polycarbonate transparent cages (33 × 13 × 14 cm) with sawdust bedding. Animals were kept on a 12 h light–dark schedule (light off from 8:00 am to 8:00 pm); the temperature was maintained at 21 °C ± 1 °C and relative humidity at 60% ± 10%. Animals were provided ad libitum with tap water and a complete pellet diet (Altromin, 1324–10 mm pellets, Lage, Germany). Since the timing of symptoms onset differs widely between the two mouse models [6,45], to test treatment efficacy on fully symptomatic mice, 5–10-month-old MeCP2-Bird and 12-month-old MeCP2-308 mice were enrolled in the present study. All procedures were carried out in accordance with the European Communities Council Directive (10/63/EU) as well as the Italian law (26/2014).

### 4.2. CNF1 Preparation and Intracerebroventricular Injection

CNF1 was obtained from the 392 *E. coli* ISS strain kindly provided by V. Falbo (Rome, Italy) and purified as previously described [47]. The recombinant protein CNF1 C866S (mCNF1), in which the enzymatic activity on Rho GTPases is abolished by a cysteine to serine substitution at amino-acid 866 [48], was used as a control. The plasmid encoding mCNF1, purified as in [47], was kindly provided by E. Lemichez (U627 INSERM, Nice, France).

Mice were anaesthetised with sevoflurane, secured to a stereotaxic apparatus, and injected at the coordinates AP 0.0, ML 0.72, and DV−2.0 relative to bregma, to target the lateral ventricles (icv), with 2 μL of CNF1 or mCNF1 solutions (10–10 M in 20 mM Tris at pH = 7.4) as in [10,11,12]. Twenty-four hours after the icv injection, mice were isolated in single cages and left undisturbed for a recovery period of one week.

### 4.3. Behavioural Testing

A battery of behavioural tests was carried out to evaluate CNF1 effects on RTT-related deficits in MeCP2-Bird and wt mice (*n* = 6–7 per experimental group; Supplementary Figure S1). Mice were experimentally naïve at the start of the behavioural test battery. All tests took place in the dark phase of the light/dark cycle, between 8.00 am and 6:00 pm, and were carried out by experimenters blind to mice genotypes and treatments.

### 4.4. Nest Building Test

Purposeful and coordinated forepaws use, a deeply compromised skill in RTT, was assessed through the nest building test, as in [25,49]. Briefly, animals were provided with one piece of filter paper each (10 × 12 cm) 3 h after the lights switched off. After 24 h, 4 and 6 days, the quality of the nests was scored by a trained observer, using the following four-point qualitative scale [50]: 0—nest material untouched; 1—nest material nearly untouched; 2—nest material scattered, no clear shape evident; 3—nest of intermediate quality; 4—nest round and well built. The nest building test was performed twice, on the 6th and 22nd days after the icv.

### 4.5. Accelerating Rotarod Test

To evaluate CNF1 effects on motor learning and coordination of experimental subjects, the accelerating rotarod test was carried out on the 9th and 26th days after the icv [51]. Each mouse was placed on the rotating rod (3 cm, Ugo Basile, Gemonio, VA, Italy), and had to walk forward in order to avoid falling. Mice underwent three consecutive trials, with an intertrial interval (ITI) of 10 min. During each trial, the rate of rotation increased from 4 to 40 r.p.m over 4 min. The trial ended when the mouse fell from the rod or remained on the rotarod for at least 270 s (cut off). The latency to fall from the rod was considered a measure of motor coordination.

### 4.6. General Health Assessment

The general health (GH) status of the mice was monitored several times throughout the experimental schedule (8, 16, 26, 37, 48 days after the icv) by a trained observer as in [52], with little modification. Each mouse received a score (in a range from 0—normal phenotype to 4—highly compromised phenotype) for all the following parameters: mobility, gait, fur, kyphosis, hindlimb clasping, tremors, breathing, presence of seizures, and general conditions. The individual scores for each parameter were subsequently averaged to obtain a semiquantitative measure of the health status, called throughout the text “GH score”.

### 4.7. Pre-Pulse Inhibition Test

Sensorimotor gating abilities of experimental mice were evaluated on the 19th day after the icv by the means of the PPI paradigm that evaluates the reduction in the acoustic startle response that occurs when presented with a pre-pulse stimulus [53]. The apparatus consisted of a Plexiglas rectangular box (startle cage, 9 × 7 cm), placed on a platform with a transducer amplifier (#PHM-250–60) inside a sound-attenuated chamber (#ENV-018S) with red lights, a fan ventilator, and an acoustic stimulator (#ANL-925, Med Associates, Inc., Albans, VT, USA). The apparatus was connected to a computer and controlled by means of a specific software (Startle Reflex Sofware, #SOF-815, Med Associates, Inc., Albans, VT, USA).

The PPI paradigm was performed as in [54] with little modification. Briefly, the day before the test, experimental animals were left undisturbed in the apparatus in the presence of the background white noise (62 dB) for 5 min, for habituation. On the test day, mice were placed in the startle chamber, where they were exposed for 5 min to the background noise, followed by three blocks of trials. The first and third blocks consisted of the presentation of 10 pulses (40 ms, 120 dB—1 pulse/trial) interspaced by an ITI of 15 s, on average. The second block consisted of a pseudorandom sequence of 56 trials, each starting with a 50 ms null period, followed by a 20 ms pre-pulse noise burst of 74, 78, 82 or 84 dB and, after a 100 ms delay, a pulse (40 ms, 120 dB). Types of trials were as follows: pre-pulse + pulse (eight trials/pre-pulse intensity), pre-pulse alone (eight trials), pulse alone (eight trials) and no stimulation (eight trials). Before each test, the apparatus was cleaned with 70% ethanol solution.

Statistical analysis was conducted separately on each of the three blocks. In the first and the third blocks the parameter measured was the ASR, measured as the mean startle amplitude for the 10 pulse alone trials. The second block was used to measure the %PPI as follows: 100 − [(mean startle amplitude for pre-pulse + pulse trials/mean startle amplitude for pulse-alone trials) × 100].

### 4.8. Fear Conditioning Test

The FC test was carried out on the 40th and 41st days after the icv to evaluate CNF1 effects on the cognitive abilities of RTT mice [10]. The apparatus consisted in a Plexiglas rectangular box (21 × 24 × 30 cm) with an electrified grid floor (conditioning chamber), inside a soundproof cubicle with red light and a speaker (Ugo Basile Fear Conditioning system, Gemonio, VA, Italy), controlled by a specific software (Anymaze Software v4.82, Stoelting, Wood Dale, IL, USA). Mice were exposed to a white noise (60 dB, 2000 Hz) throughout the task.

The FC protocol took place in two consecutive days, as previously described [10]. On the first day (training phase), mice were placed in the conditioning chamber for a 3-min-acclimation period (baseline, BL). Afterwards, they were exposed to three 30 s acoustic stimuli (80 dB, 2000 Hz-conditioned stimulus, CS) paired, during the last 2 s, with an electric foot-shock (0.7 mA), and spaced by an ITI of 30 s. On the second day (test phase), mice were first re-exposed to the conditioning context for 3 min (contextual fear) and then subjected to 15 trials of 30 s-CS exposure, with an ITI of 10 s (cued fear). Before the start of each session, the grid floor of the apparatus was cleaned with 70% ethanol. The time spent freezing, described as the absence of body activities except for respiratory-related movements, was measured by an automatic freezing detector (Anymaze Software v4.82, Stoelting, Wood Dale, IL, USA), and considered a measure of fear. As an index of contextual and cued fear memories, the Δ freezing was obtained by subtracting, in each subject, the percent time spent freezing during baseline from the percent time spent freezing during either context or CS re-exposure, respectively.

### 4.9. Mitochondrial Analyses

To verify MRC activities in MeCP2-Bird mouse brain and CNF1 treatment effects thereon, on the 50th day after the icv mice were sacrificed, the brains were dissected, and cortices were immediately frozen in dry-ice, and stored at −80 °C (*n* = 3–4 per group). Mitochondria were isolated by differential centrifugation of brain homogenate as previously described [55]. Briefly, mitochondrial pellets were suspended in 1 mL of 10 mM Tris-HCl (pH = 7.5) plus 1 mg/mL BSA and exposed to ultrasound energy for 8 s at 0 °C (11 pulses 0.7 s on, 0.7 s off) at 20 kHz, intensity 2. The ultrasound-treated mitochondria were centrifuged at 600× *g* for 10 min, 4 °C. The supernatant was centrifuged again at 14,000× *g* for 10 min at 4 °C and the resulting pellet was kept at −80 °C until use. MRC complex activities were performed, essentially as in [26] by two assays relying on the sequential addition of reagents to measure (i) the activity of complex I as rotenone-sensitive NADH:ubiquinone oxidoreductase, followed by the oligomycin-sensitive ATPase activity of complex V; (ii) the activity of complex II as malonate-sensitive succinate:ubiquinone oxidoreductase.

### 4.10. Western Blotting

To analyse CNF1 treatment effects at the molecular level, the hippocampus, a brain area particularly involved in RTT pathophysiology, was dissected, immediately frozen in dry-ice, and stored at −80 °C. The hippocampus was also collected from a separate cohort of MeCP2-308 female mice, previously subjected to a comparable battery of behavioural tests and sacrificed 35 days after the icv injection. Tissues were lysed in RIPA buffer (50 mM Tris-HCl, pH = 7.4, 150 mM NaCl, 1% NP-40, 2 mM EDTA plus 10 µg/mL aprotinin, 10 µg/mL leupeptin, 1 mM PMSF, 1 mM Na_2_VO_4_) at +4 °C according to standard procedures. Twenty-five total protein extracts were resolved on 12% SDS/PAGE and electrically transferred onto poly (vinylidene difluoride) membranes (Bio-Rad Laboratories, Hercules, CA, USA). Membranes were blocked with TBS-T (20 mM Tris/HCl, pH = 7.4, 150 mM NaCl, 0.02% Tween-20) containing 5% skimmed milk (Bio-Rad Laboratories, Hercules, CA, USA) for 1 h at room temperature and then incubated overnight at 4 °C with the following primary antibodies diluted in TBS-T containing 5% milk or 5% BSA (Sigma-Aldrich, Saint Louis, MO, USA): rabbit monoclonal anti-Tom20 (1:1000; Cell Signaling, Danvers, MA, USA), rabbit polyclonal anti-spinophilin (1:1000; Millipore, Burlington, MA, USA), rabbit monoclonal mTOR, phosphor-mTOR^Ser2448^ (1:1000 in BSA; Cell Signaling, Danvers, MA, USA), and mouse monoclonal anti-GAPDH (1:1000; Santa Cruz Biotechnology, Dallas, TX, USA). After thorough washing in TBS-T, immunocomplexes were revealed with horseradish-peroxidase-conjugated species-specific secondary antibodies (Jackson Laboratory, Bar Harbor, ME, USA) followed by an enhanced chemiluminescence reaction (Millipore Corporation, Darmstadt, Germany). Reactive bands were detected by the ChemiDoc MP system (Bio-Rad Laboratories, Hercules, CA, USA) and signal quantification was performed using the IMAGE LAB software 5.0 (Bio-Rad Laboratories, Hercules, CA, USA). Briefly, we chose images not overexposed and we performed a densitometric analysis of reactive bands that we defined, after background subtraction. The arbitrary units obtained were used to calculate the relative increase/decrease of bands.

### 4.11. Statistical Analyses

Statistical analyses were performed using Statview Software v.5.0.1 (Abacus Concept, Berkeley, CA, USA). Data were analysed using two-way analysis of variance (ANOVA), with genotype and treatment as between-subject factors, or applying three-way mixed ANOVA, setting repeated measurements as within-subject factors*. Post hoc* comparisons were performed using Tukey’s test [56]. Animals identified as outliers by the use of Grubb’s test were excluded from the analyses [57]. A probability level of *p* < 0.05 was considered to be statistically significant. Results are presented as mean ± standard error of the mean (SEM). All statistical analyses were performed by experimenters blind to animals’ genotype and treatment.

## 5. Conclusions

In conclusion, the present study provides evidence that the CNF1 treatment may provide differential outcomes for RTT depending on the MeCP2 mutation. Taken together these results underline the importance of exploiting the available RTT mouse models carrying different MeCP2 mutations for testing the therapeutic efficacy of novel therapeutic approaches.

## Data Availability

Data used in this article are available upon request to the corresponding author.

## Supplementary Materials

The following are available online at https://www.mdpi.com/article/10.3390/ijms22136739/s1, Figures S1–S5.
