# Supplementary material for: Treatment with the Bacterial Toxin CNF1 Selectively Rescues Cognitive and Brain Mitochondrial Deficits in a Female Mouse Model of Rett Syndrome Carrying a MeCP2-Null Mutation
Source: Int J Mol Sci. 2021 Jun 23;22(13):6739. doi: 10.3390/ijms22136739 (PMC8269120; doi:10.3390/ijms22136739)
Supplement: Supplementary file 1 [file ijms-22-06739-s001.zip › ijms-1226010-SI.pdf]

Supplement to:

Treatment with the Bacterial Toxin CNF1 Selectively Rescues Cognitive and Brain Mitochondrial Deficits in a Female Mouse Model of Rett Syndrome Carrying a MeCP2-Null Mutation

Supplementary Materials

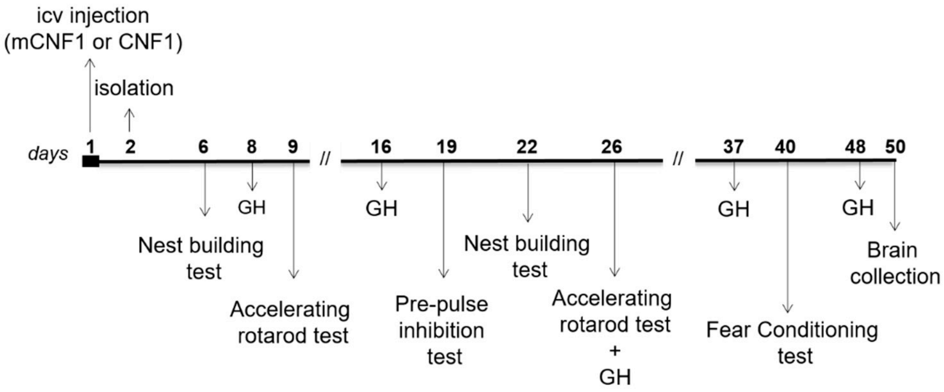

Figure S1. Timeline of the experimental schedule. MeCP2-Bird mice and wild type littermates were intracerebroventricularly (icv) injected with either CNF1 or the recombinant protein CNF1 C866S (mCNF1) as control. Twenty-four hours after the icv injection, mice were isolated and left undisturbed for one week. Subsequently, a battery of behavioural tests including nest building test, accelerating rotarod test, pre-pulse inhibition test and fear conditioning test, was carried out to evaluate CNF1 treatment on RTT-related behavioural alterations. Throughout the experimental schedule, treatment effects on the general health (GH) status of mice were also assessed. At the end of behavioural testing, 50 days after the icv injection, mouse brains were collected and dissected for biochemical and molecular analyses.

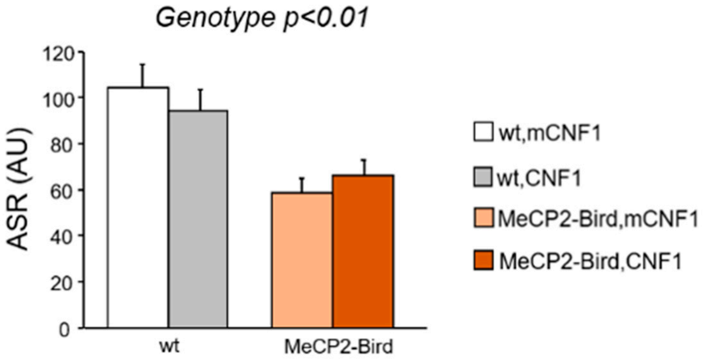

Figure S2. Treatment with CNF1 does not affect the acoustic startle response (ASR) of MeCP2-Bird mice. The ASR was measured from the first and third blocks of trials, as the mean startle amplitude for pulse alone trials (measured in average units, AU). The main effect of genotype was found with MeCP2-Bird female mice treated with an inactive form of the bacterial toxin (mCNF1) showing reduced ASR respect to wild type (wt) mice. CNF1 treatment did not normalise this parameter. Mice for each condition were as follows: wt, mCNF1: 7; wt, CNF1: 7; MeCP2-Bird, mCNF1: 6; MeCP2-Bird, CNF1: 6. Data are mean ± SEM. Statistical significance was assessed using three-way mixed ANOVA.

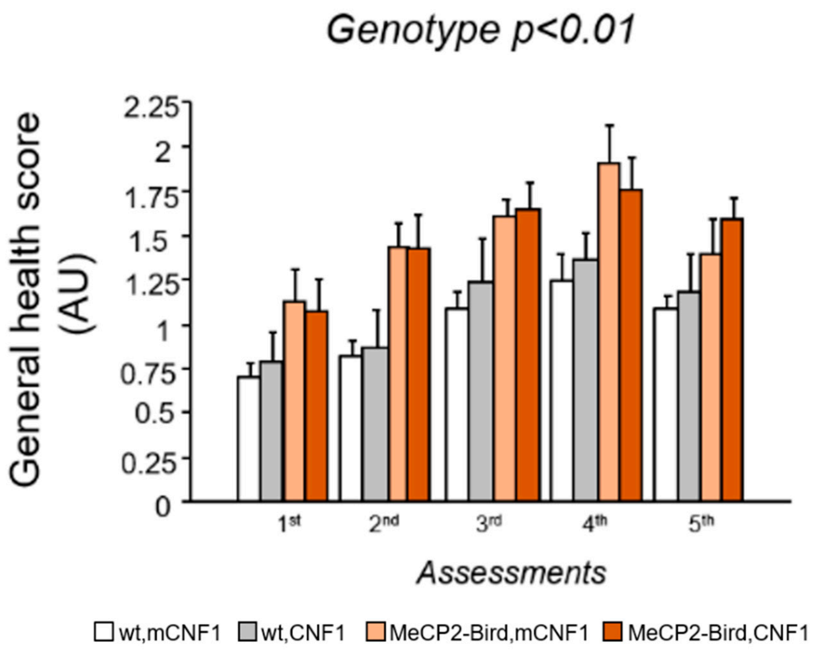

Figure S3. CNF1 treatment does not rescue the general health impairments of symptomatic MeCP2-Bird female mice. The general health of experimental animals was qualitatively evaluated by a trained observer, blind to mouse genotype and treatment, five times throughout the experimental schedule and measured in average units (AU). The impaired health conditions showed by symptomatic MeCP2-Bird females treated with an inactive form of the bacterial toxin (mCNF1) were not rescued by CNF1 administration. Mice for each condition were as follows: wt, mCNF1: 7; wt, CNF1: 7; MeCP2-Bird, mCNF1: 6; MeCP2-Bird, CNF1: 6. Data are mean ± SEM. Statistical significance was assessed using three-way mixed ANOVA.

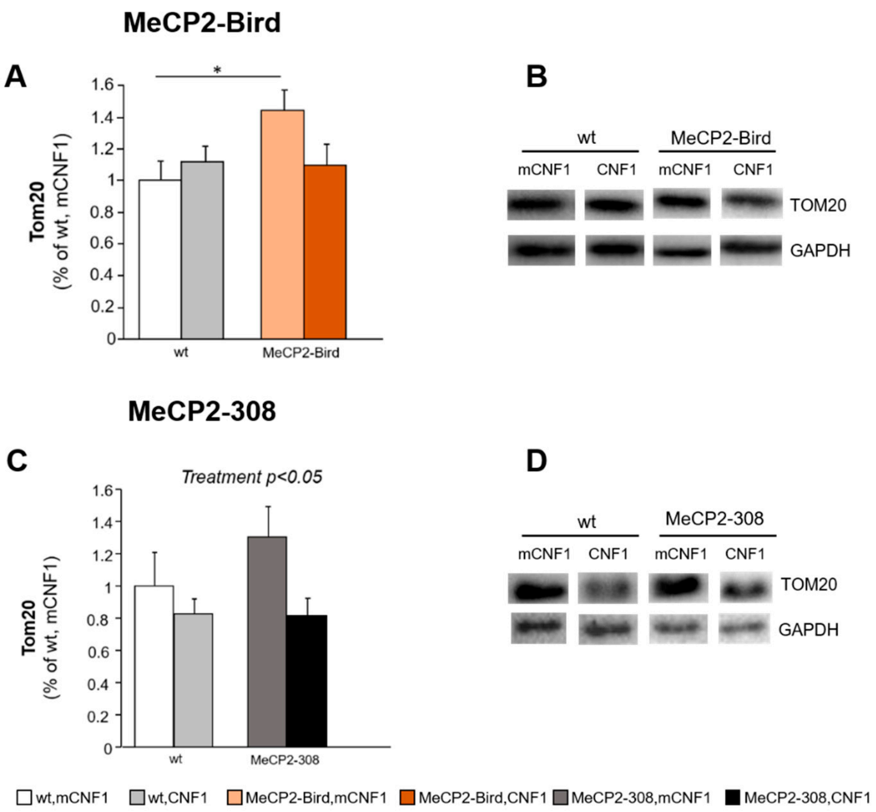

Figure S4. Treatment with CNF1 does not affect the expression of a mitochondrial marker in MeCP2-Bird and MeCP2-308 symptomatic mice. Expression of the mitochondrial

protein Tom20, involved in the mitochondrial translocation of nuclear-encoded proteins, was evaluated by the means of Western blot analyses in hippocampi dissected from the brains of MeCP2-Bird, MeCP2-308 mice, and wild type (wt) littermates treated with the recombinant (inactive, mCNF1) or active form of the bacterial toxin CNF1. (**A**,**C**) Tom20, a central component of the translocase of the outer membrane mitochondrial receptor complex, was significantly increased in MeCP2-Bird, mCNF1 mice compared to wt controls, but treatment with CNF1 failed to significantly modulate its levels in MeCP2-Bird hippocampi. No significant differences were found in Tom20 expression between MeCP2-308 and wt littermates, and CNF1 treatment significantly reduced Tom20 levels. (**B**,**D**) Immunoblots are examples from one animal of each experimental group. Tom20 levels are normalised to total glyceraldehyde 3-phosphate dehydrogenase (GAPDH) contents and expressed as a proportion of those of wt, mCNF1 mice. Mice for each condition were as follows: wt, mCNF1: 12 and 9; wt, CNF1: 10 and 8; MeCP2-Bird, mCNF1: 12; MeCP2-Bird, CNF1: 12; MeCP2-308, mCNF1: 8; MeCP2-308, CNF1: 9. Data are mean ± SEM. Statistical significance was assessed using two-way ANOVA and Tukey's *post hoc* tests. *: $p < 0.05$.

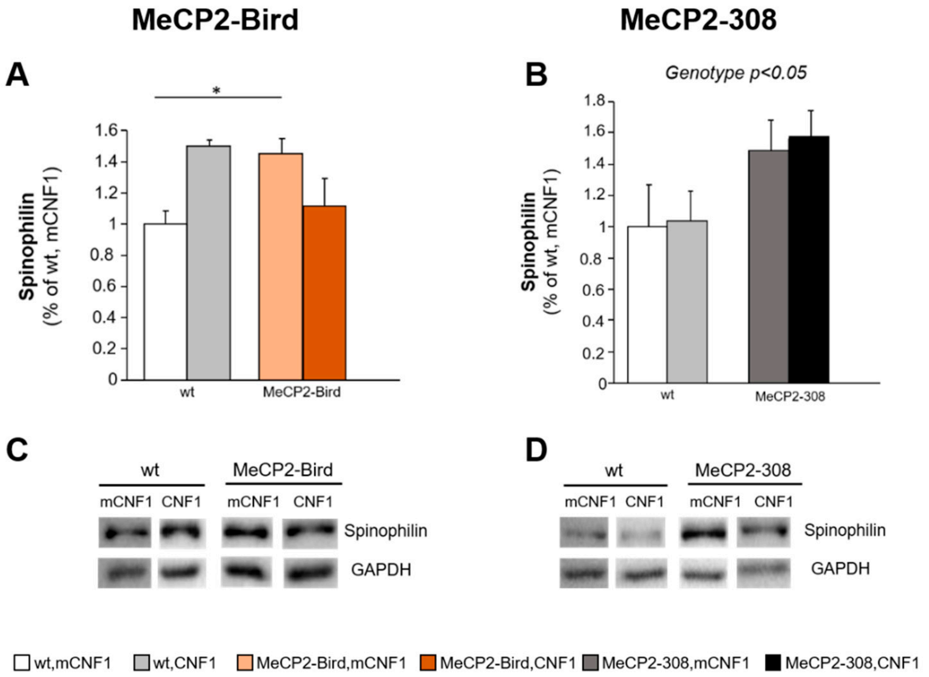

Figure S5. Treatment with CNF1 does not normalise the expression of the synaptic protein spinophilin, that is increased in both MeCP2-Bird and MeCP2-308 symptomatic mice. Spinophilin expression was evaluated by the means of Western blot analyses in hippocampi dissected from the brains of MeCP2-Bird, MeCP2-308 mice, and wild type (wt) littermates treated with the recombinant (inactive, mCNF1) or active form of the bacterial toxin CNF1. (**A**,**B**) Both strains showed significantly higher levels of spinophilin in the hippocampus compared to wt controls. Treatment with CNF1 did not normalise this phenotype in either strain. (**C**,**D**) Immunoblots are examples from one animal of each experimental group. Spinophilin levels are normalised to total glyceraldehyde3-phosphate dehydrogenase (GAPDH) contents and expressed as a proportion of those of wt, mCNF1 mice. Mice for each condition were as follows: wt, mCNF1: 6 and 7; wt, CNF1: 4 and 7; MeCP2-Bird, mCNF1: 6; MeCP2-Bird, CNF1: 6; MeCP2-308, mCNF1: 7; MeCP2-308, CNF1: 7. Data are mean ± SEM. Statistical significance was assessed using two-way ANOVA and Tukey's *post hoc* tests. *: $p < 0.05$.
